# Supplementary material for: Implant-to-implant wireless networking with metamaterial textiles
Source: Nat Commun. 2023 Jul 19;14:4335. doi: 10.1038/s41467-023-39850-2 (PMC10356940; doi:10.1038/s41467-023-39850-2)
Supplement: Supplementary file 1 — Supplementary Information [file 41467_2023_39850_MOESM1_ESM.pdf]

Supplementary: Implant-to-implant wireless networking with metamaterial textiles

Xi Tian*†,[1,2] Qihang Zeng†,[1,2] Selman A. Kurt†,[1] Renee R. Li,[3,4] Dat T. Nguyen,[5] Ze Xiong,[1,2,6,7] Zhipeng Li,[1] Xin Yang,[1] Xiao Xiao,[1] Changsheng Wu,[2,6,8] Benjamin C. K. Tee,[2,6,8] Denys Nikolayev,[9] Christopher J. Charles,[3,4,10] and John S. Ho*[1,2,6]

[1] *Department of Electrical and Computer Engineering, National University of Singapore, Singapore 117583, Singapore*

[2] *Institute for Health Innovation and Technology, National University of Singapore, Singapore 117599, Singapore*

[3] *Cardiovascular Research Institute, National University Heart Centre, Singapore 117599, Singapore*

[4] *Department of Surgery, Yong Loo Lin School of Medicine, National University of Singapore, Singapore 119228, Singapore*

[5] *Integrative Sciences and Engineering Program, NUS Graduate School, National University of Singapore, Singapore 119077, Singapore*

[6] *The N.1 Institute for Health, National University of Singapore, Singapore 117456, Singapore*

[7] *Department of Biomedical Engineering, National University of Singapore, Singapore 117583, Singapore*

[8] *Department of Materials Science and Engineering, National University of Singapore, Singapore 117575, Singapore*

[9] *IETR – UMR 6164, CNRS, University of Rennes 1, Rennes, France*

[10] *Christchurch Heart Institute, Department of Medicine, University of Otago, Christchurch, New Zealand*

* Corresponding authors. E-mails: tianxi@u.nus.edu; johnho@nus.edu.sg.

† These authors contributed equally to this work.

SUPPLEMENTARY METHODS

I. THEORETICAL ANALYSIS

A. Phased surface design

The focusing effect of the side-fed phased surface results from the combination of radiation from currents oscillating with different phases in the annular rings. The desired amplitudes and phases are achieved by solving corresponding passive components using multiport optimization methods[1]. The multiport network consists of the side-fed port of the phased surface (labeled Port 0), the terminals loaded with passive components (labeled Port 1 to 9), and the dipole source (Port 10) placed at the focusing point parallel to the input port. CST Microwave Studio is used to acquire the impedance matrix for the multiport network by placing the side-fed phased surface on a homogenous tissue volume. The impedance matrix for the 11-port structure is given by:

$$\vec{v} = Z\vec{i} \tag{1}$$

To further compute the efficiency of focusing performed by the fields in tissue, the receiver port is isolated by partitioning the impedance matrix. The multiport network satisfies the following set of balance equations:

$$\begin{pmatrix} V_s \\ 0 \end{pmatrix} = \begin{pmatrix} Z_s & Z_m \\ Z_m^T & z_r + z_l \end{pmatrix} \begin{pmatrix} I_s \\ i_r \end{pmatrix} \tag{2}$$

where V_s (I_s) is the source voltage (current) vector, Z_s is the upper-left block of the Z matrix, Z_m is the mutual impedance vector between the source and receiver ports, z_r is the self-impedance of the receiver, and z_l is the load-impedance of the receiver port. The Schur complement of the block matrix is as follows:

$$S := Z_s - \frac{Z_m Z_m^T}{z_r + z_l} \tag{3}$$

Thus, after elimination of the receiver port, the multiport network matrix can be reduced as:

$$V_s = S I_s \tag{4}$$

The transmitted power is given by:

$$P_t = \frac{\mathrm{Re}(I_s^H V_s)}{2} \tag{5}$$

The power received is given by:

$$P_r = \frac{\mathrm{Re}(i_r^* v_r)}{2} \tag{6}$$

Thus, the transfer efficiency of the system is given by:

$$\eta := \frac{P_r}{P_t} = \frac{\mathrm{Re}(z_l)}{\mid z_r + z_l \mid^2} \frac{\mid Z_m^T I_s \mid^2}{I_s^H [\mathrm{Re}(S)] I_s} \tag{7}$$

To maximize the efficiency, the optimization problem need to be solved:

$$\max_{I_s} \quad \frac{I_s^H Z_m^* Z_m^T I_s}{I_s^H [\mathrm{Re}(S)] I_s} \tag{8}$$

The solution to such a generalized eigenvalue problem with unity rank matrix $Z_m^* Z_m^T$ is given by:

$$I_s^{\mathrm{opt}} = [\mathrm{Re}(S)]^{-1} Z_m^* \tag{9}$$

Reactive components are used to implement the above "matched filter"[2,3]. To solve the required reactive loading values, the symmetric S matrix can be partitioned by isolating the active side-fed port:

$$\begin{pmatrix} v_0 \\ V_{sp} \end{pmatrix} = \begin{pmatrix} s_0 & S_m^T \\ S_m & S_p \end{pmatrix} \begin{pmatrix} i_0 \\ I_{sp} \end{pmatrix} \tag{10}$$

where i_0 is current flow of the active side-fed port (Port 0). This yields another set of equations:

$$V_{sp} = -Z_{sp} I_{sp} \tag{11}$$

where Z_{sp} is a diagonal matrix:

$$Z_{sp} = \begin{pmatrix} z_{s,1} & & \\ & \ddots & \\ & & z_{s,9} \end{pmatrix} \tag{12}$$

In order to achieve I_s^{opt}, the diagonal entries $z_{s,n}$ are set to

$$z_{s,n} = -\frac{i_0^{\mathrm{opt}} S_{m,n} + (S_p I_{sp}^{\mathrm{opt}})_n}{I_{sp,n}^{\mathrm{opt}}} \tag{13}$$

The obtained reactive (imaginary) impedance values could be inductive or capacitive. The resistance (real) values are ignored for simplicity. The passive component values are assigned according to the reactances. Here, from the outermost ring to the center, the reactances are found to be all capacitive and the closest available commercial components are chosen (Table I).

B. Spoof surface plasmonic (SSP) waveguide design

The SSP waveguide is designed by determining geometrical parameters to support surface-plasmon-like modes with desired properties over the $2.4 - 2.5$ GHz ISM frequency band[4,5]. The design process utilizes a simplified human body model consisting of a half-space filled with free-space in the upper region ($z > 0$) and a biological tissue region of dielectric permittivity ε_r in the lower region($z < 0$). The SSP waveguide is placed on the interface ($z = 0$) and supports surface modes propagating in the $\pm x$ directions. The electric field can be approximated by

$$\mathbf{E}_{\pm}(\mathbf{r}) = p(y) \begin{pmatrix} i\alpha_n/\beta \\ 0 \\ 1 \end{pmatrix} e^{\pm i\beta x - \alpha_n |z|} \tag{14}$$

where $p(y)$ represents the field profile in the y direction, β the propagation constant, and α_n the decay constant in each region. In addition to support surface-plasmon-like dispersion relations, the propagation constant β follows the surface plasmon dispersion of the form

$$\beta = k_0 \sqrt{\varepsilon_1 \varepsilon_2 / (\varepsilon_1 + \varepsilon_2)} \tag{15}$$

where $k_0 = \omega/c$ is the wavenumber, ε_1 is the dielectric permittivity, and $\varepsilon_2 = 1 - \omega_p^2/\omega^2$. The dispersion curve lies right to the light line ($\beta = k_0$) approaches a horizontal asymptote at the surface plasma frequency $\omega_{sp} = \omega_p/\sqrt{1+\varepsilon_1}$. The decay constant for the upper free-space regions is given by $\alpha_1 = \sqrt{\beta^2 - k_0^2}$, which implies that the mode is confined to the surface of the upper region because α_1 is purely real. In the lower region, the field is screened by an additional unpatterned bottom layer such that $\alpha_2 \to \infty$ to prevent the additional radiative modes in the body region $\beta < \omega\sqrt{\varepsilon_\mathrm{body}}/c$. To acquire the desired geometrical parameters, numerical simulations are conducted to obtain the surface mode characteristics. Eigenmode analysis using CST Microwave Studio (Dassault Systems) reveals tunable surface-plasmon-like dispersion curves through geometrical parameters adjustment.

II. RECEIVED POWER MEASUREMENT

Field intensity measurements used an RF magnetic field probe (Langer EMW-Technik, RF R 0,3-3) mounted on a two-axis positioning system (HANPOSE, 17HS3401S). The received power from the probe is monitored by a spectrum analyzer (Keysight CXA Signal Analyzer) at each position. Step sizes of 2 mm were used. Fields were generated in a water container filled with water by the metamaterial textile conformally attached to the wall at a 15 cm height above the floor.

III. IMPLANT NODES ENCAPSULATION

The implant devices are encapsulated by pouring polydimethylsiloxane (PDMS) over a 3D-printed mould (ABS filament). PDMS is prepared by mixing an elastomer base (Sylgard, 3097366-1004) with a silicone elastomer curing agent (Sylgard, 3097358-1004) at a 10:1 ratio for 15 minutes. The mixture is degassed in a vacuum chamber, and then cured in an oven at 70° overnight.

IV. IMAGING

Computed tomography (256 Flash CT scanner, Siemens) was performed on a pig carcass (female, 45kg) in the National Large Animals Research Facility (NLARF). The scan parameters were 100 kVp, 179 mAs, helical. The slice thickness was 0.5 mm in all scans.

SUPPLEMENTARY REFERENCES

[1] D. R. Agrawal, Y. Tanabe, D. Weng, A. Ma, S. Hsu, S.-Y. Liao, Z. Zhen, Z.-Y. Zhu, C. Sun, Z. Dong, F. Yang, H. F. Tse, A. S. Y. Poon, and J. S. Ho, "Conformal phased surfaces for wireless powering of bioelectronic microdevices," Nature Biomedical Engineering **1**, 0043 (2017).

[2] R. Harrington, "Reactively controlled directive arrays," IEEE Transactions on antennas and propagation **26**, 390–395 (1978).

[3] J. S. Ho, B. Qiu, Y. Tanabe, A. J. Yeh, S. Fan, and A. S. Poon, "Planar immersion lens with metasurfaces," Physical Review B **91**, 125145 (2015).

[4]X. Tian, P. M. Lee, Y. J. Tan, T. L. Wu, H. Yao, M. Zhang, Z. Li, K. A. Ng, B. C. Tee, and J. S. Ho, "Wireless body sensor networks based on metamaterial textiles," Nature Electronics **2**, 243–251 (2019).

[5]X. Tian, Q. Zeng, D. Nikolayev, and J. S. Ho, "Conformal propagation and near-omnidirectional radiation with surface plasmonic clothing," IEEE Transactions on Antennas and Propagation **68**, 7309–7319 (2020).

SUPPLEMENTARY FIGURES

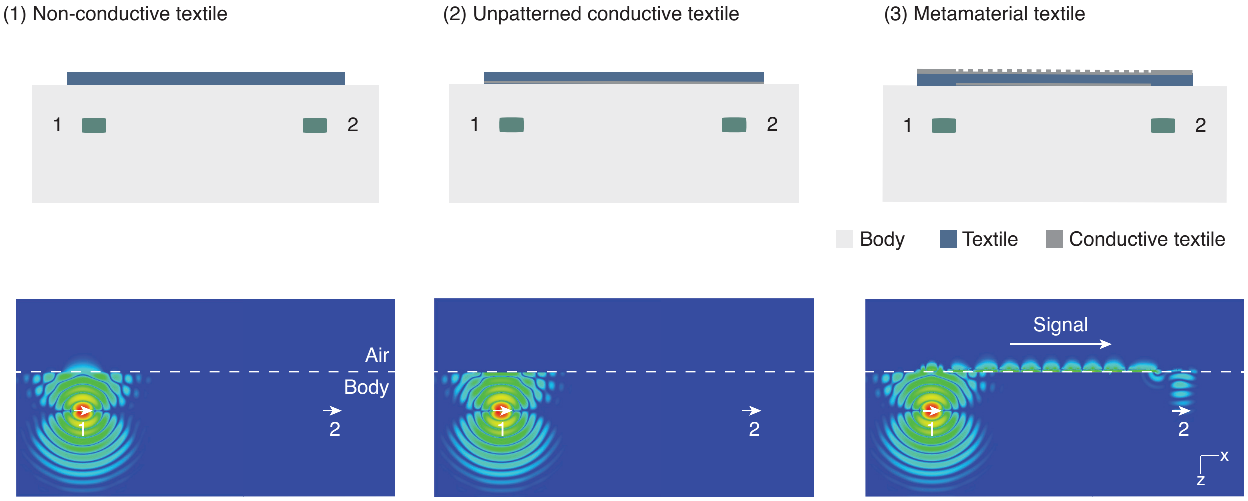

Supplementary Figure 1. Full-wave simulation of wireless transmission of implanted antennas. Comparisons of simulated magnetic field distribution of the (1) non-conductive textile, (2) unpatterned conductive textile, and (3) metamaterial textile.

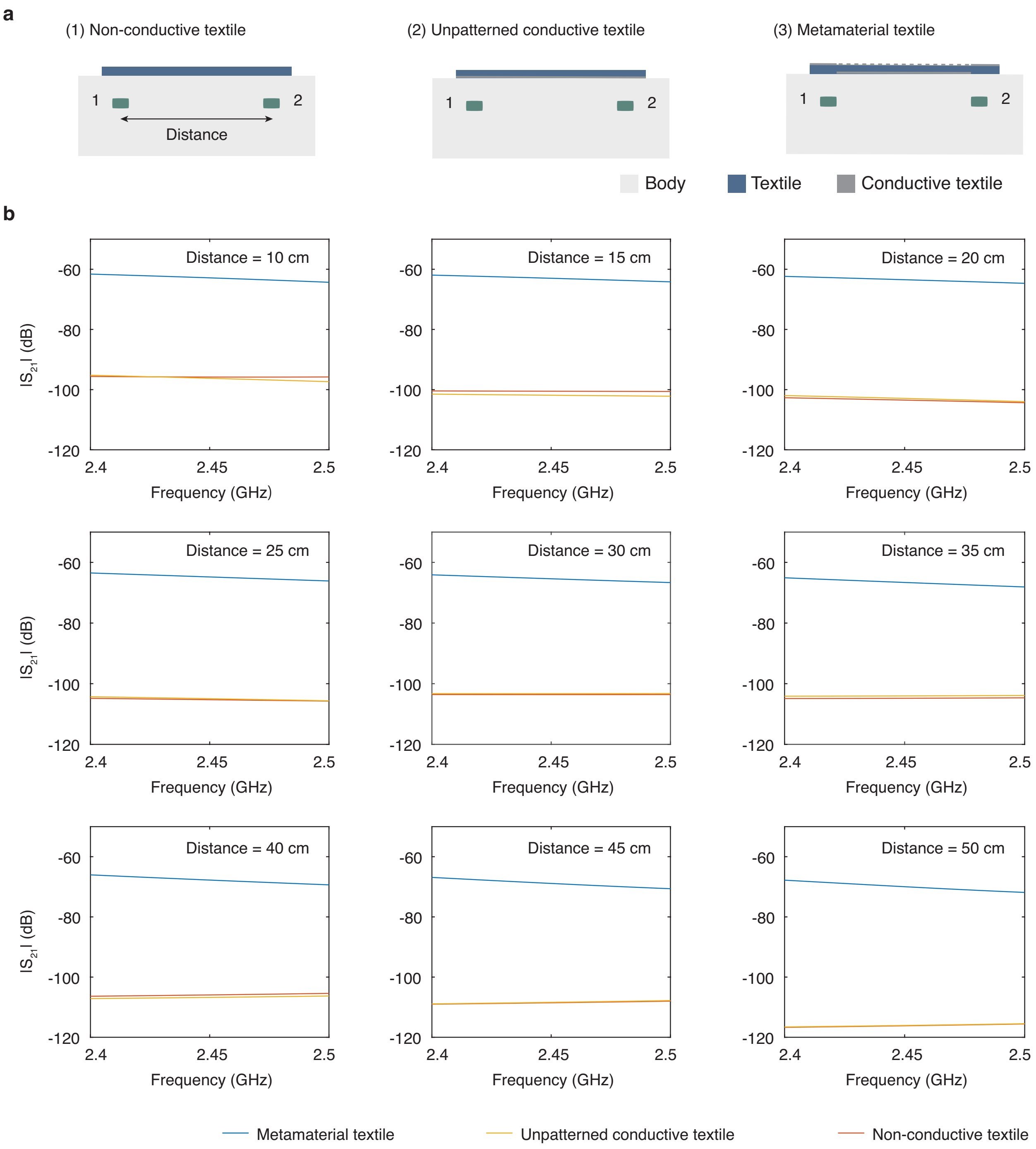

Supplementary Figure 2. Comparison of transmission spectrums. a, Illustration of wireless implant-to-implant communication comparisons. **b,** Transmission spectrum $|S_{21}|$ for the (1) non-conductive textile, (2) unpatterned conductive textile, and (3) metamaterial textile with different lengths.

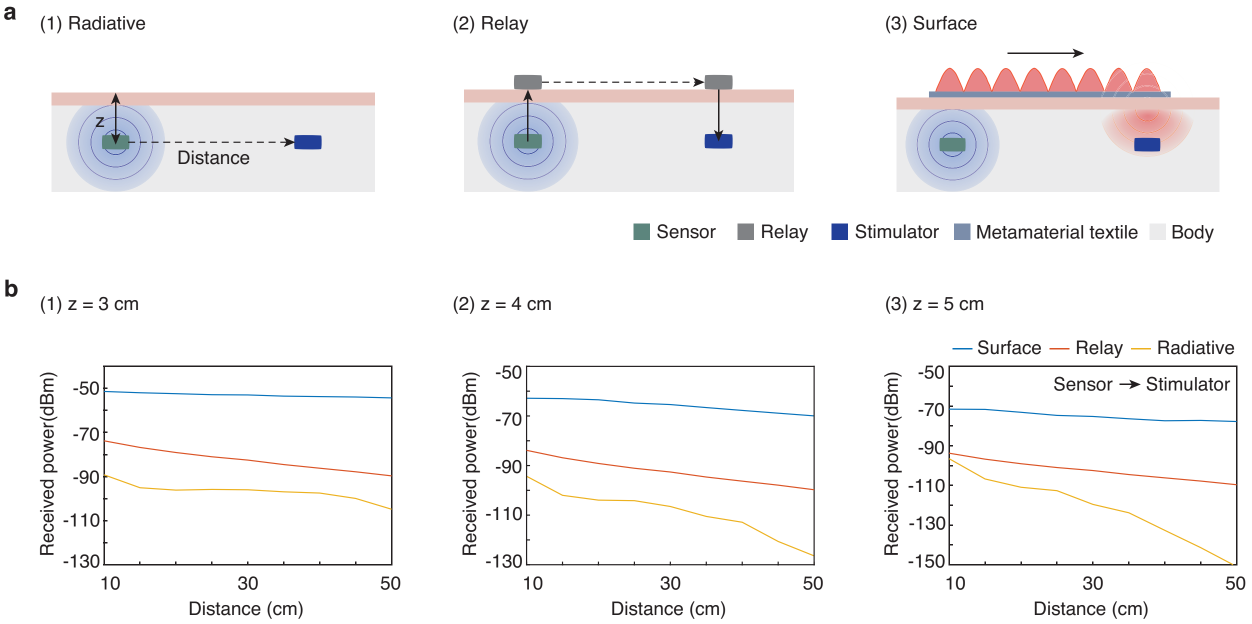

Supplementary Figure 3. Comparison of the transmission coefficient as a function of distance. a, Illustrations of different wireless implant-to-implant communication methods. **b,** Received power as a function of distance. The devices are implanted in varied depth (z), from 3 to 5cm.

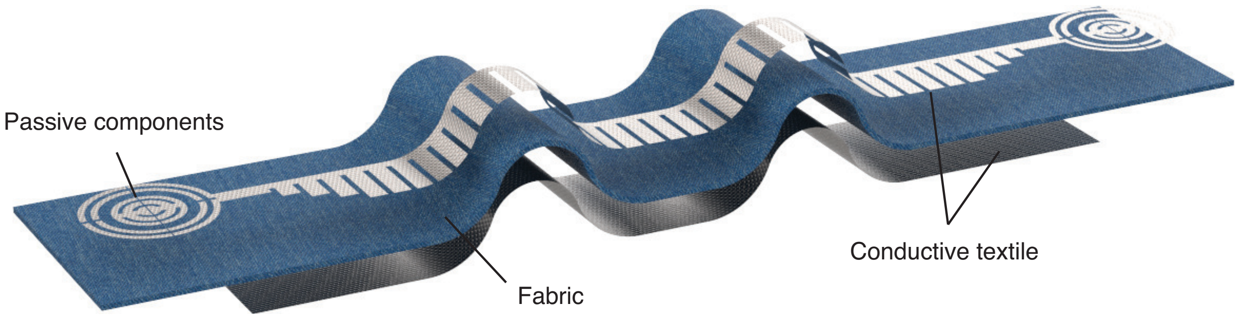

Supplementary Figure 4. Structure of the metamaterial textile. Schematic of the creased metamaterial textile consists of a patterned top layer, an intermediate fabric layer, and a bottom ground plane.

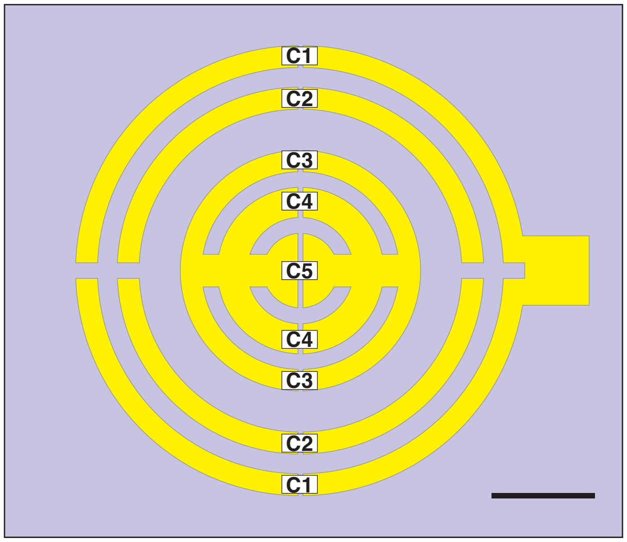

Supplementary Figure 5. Structure of the side-fed phased surface. All dimensions are to scale. Passive components are listed in Supplementary Table I. Scale bar, 1 cm.

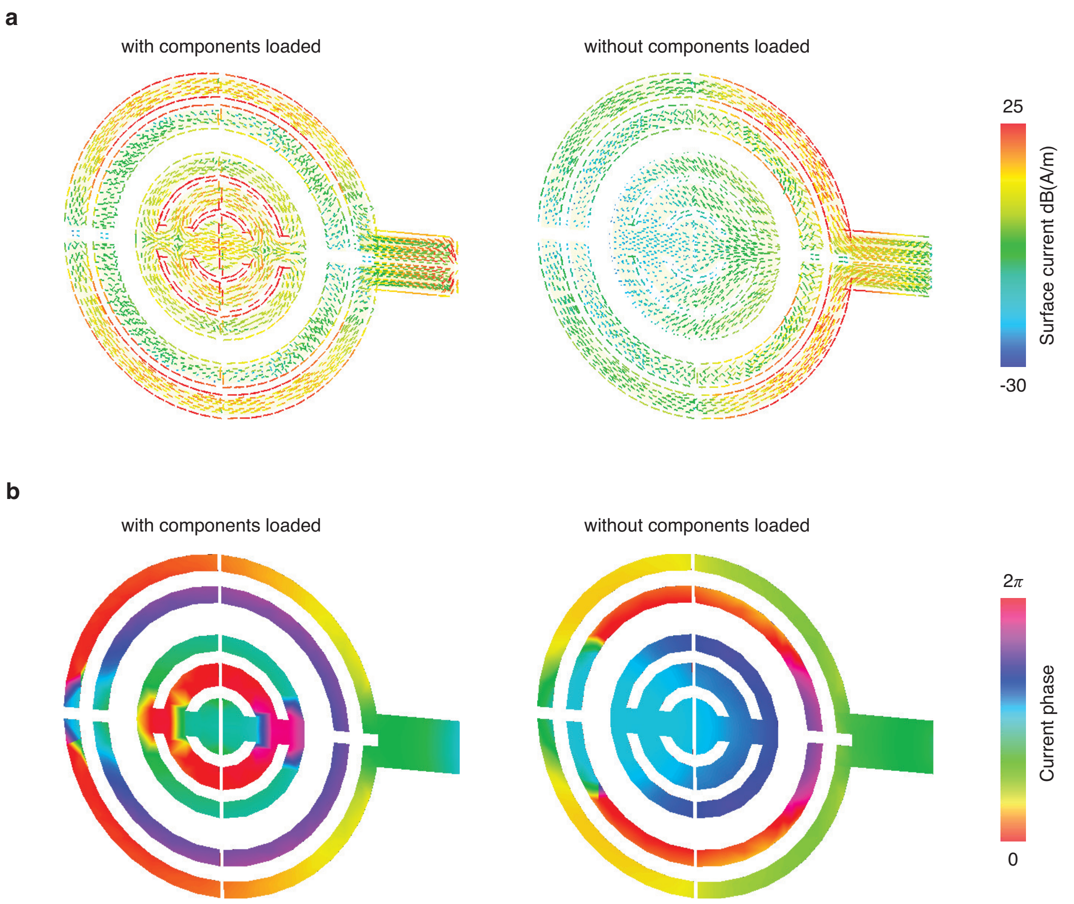

Supplementary Figure 6. Comparison of surface current and phase distribution of the side-fed phased surface. Instantaneous surface current vector distribution (**a**) and phase difference (**b**) during continuous-wave excitation with and without passive components loaded.

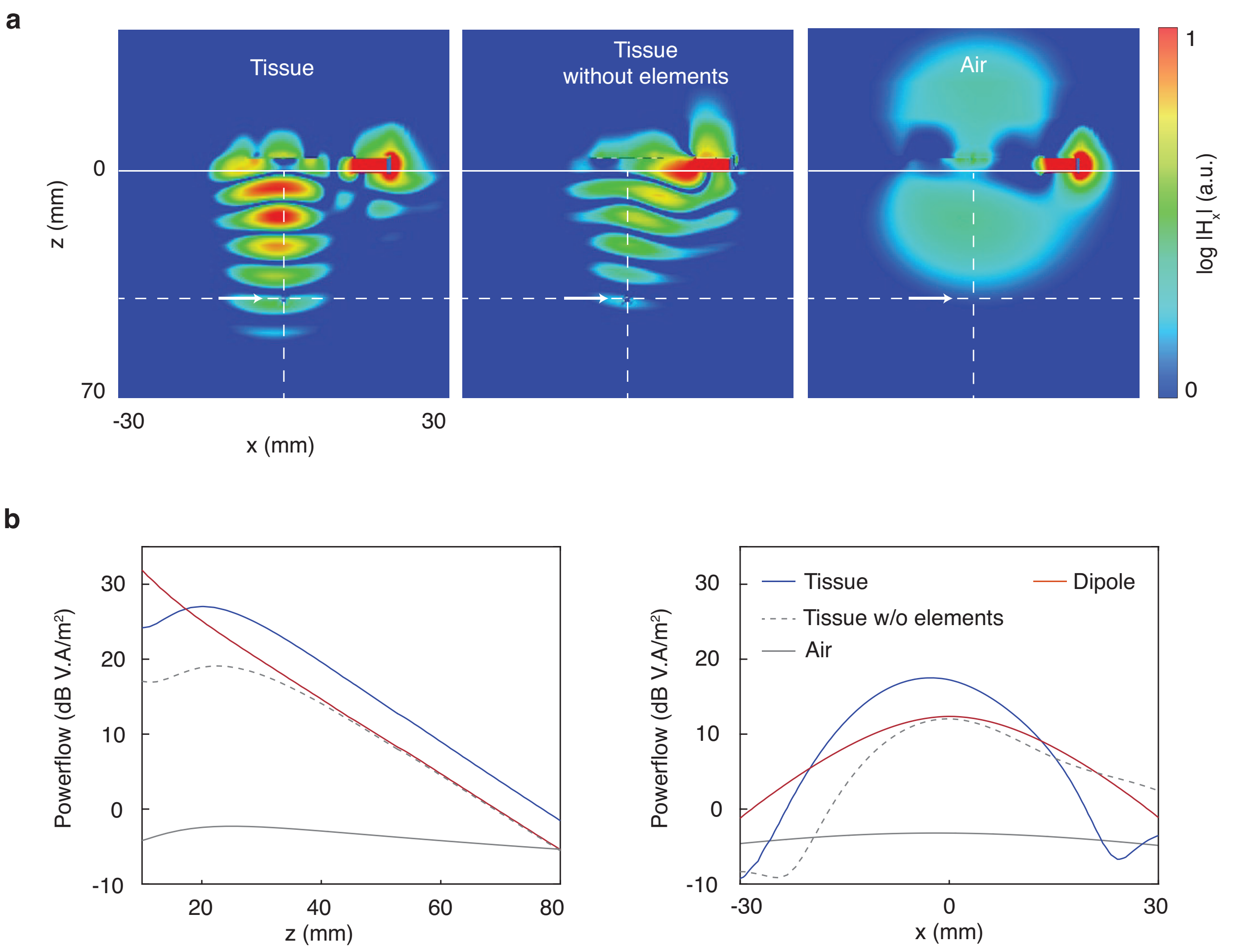

Supplementary Figure 7. Wireless communication performance of the side-fed phased surface. a, Simulated magnetic field intensity generated by the side-fed phased surface above tissue, tissue without reactive loading elements and in air. The receiver antenna is placed at a 4 cm depth (white arrow). **b,** Comparison of simulated powerflow of the side-fed phased surface with a dipole antenna along the vertical and horizontal white dashed lines in (**a**).

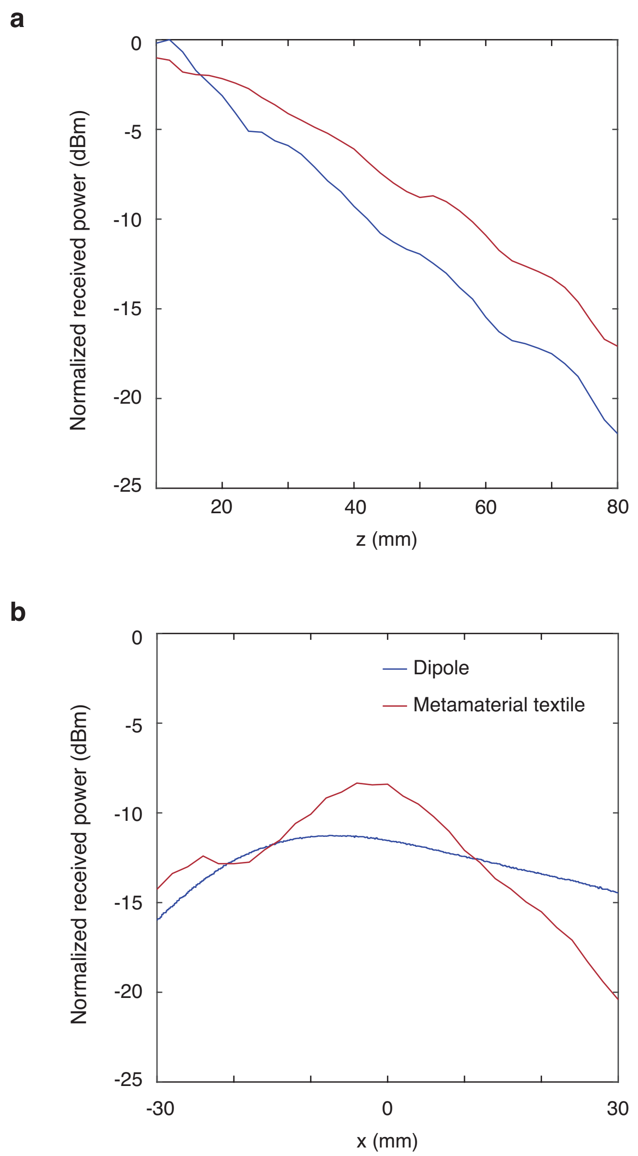

Supplementary Figure 8. Wireless communication performance in water. Comparison of measured magnetic field intensity of the metamaterial textile with a dipole antenna attached to the wall of the water container. **a,** Normalized received power as a function of depth (z). **b,** The measured received power in the water at a depth of 4 cm.

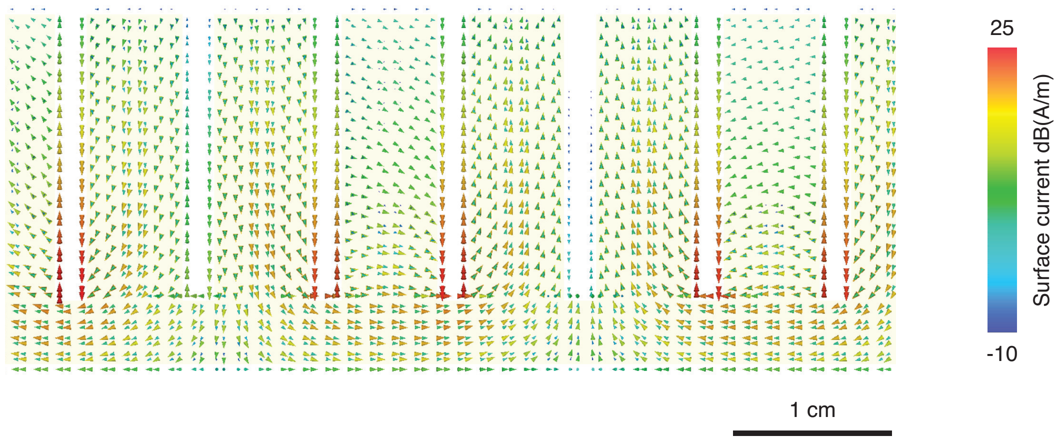

Supplementary Figure 9. Surface current of the SSP waveguide. The simulated surface current vector distributions of the spoof surface plasmonic mode.

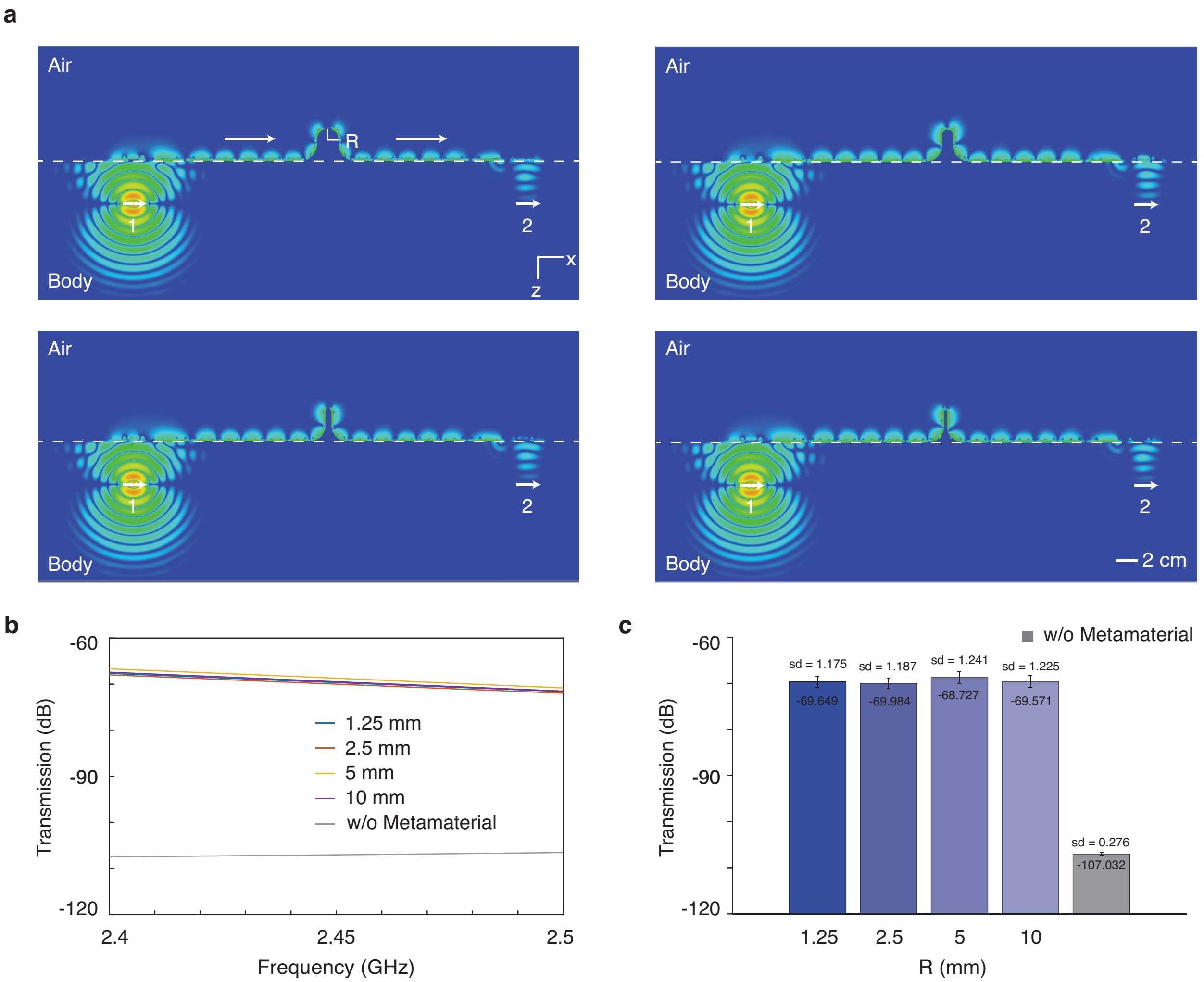

Supplementary Figure 10. Propagation on folded metamaterial textiles. a, Full-wave simulations of the magnetic field (xz plane) at 2.4 GHz for creased metamaterial textiles with varying radii-of-curvature R. **b,** Transmission coefficient as a function of numbers of creases. **c,** Transmission (S_{21}) as a function of R. The Grey bar shows transmission measured without the metamaterial textile. Error bars show mean ± s.d. of the transmission spectra in 2.4–2.5 GHz.

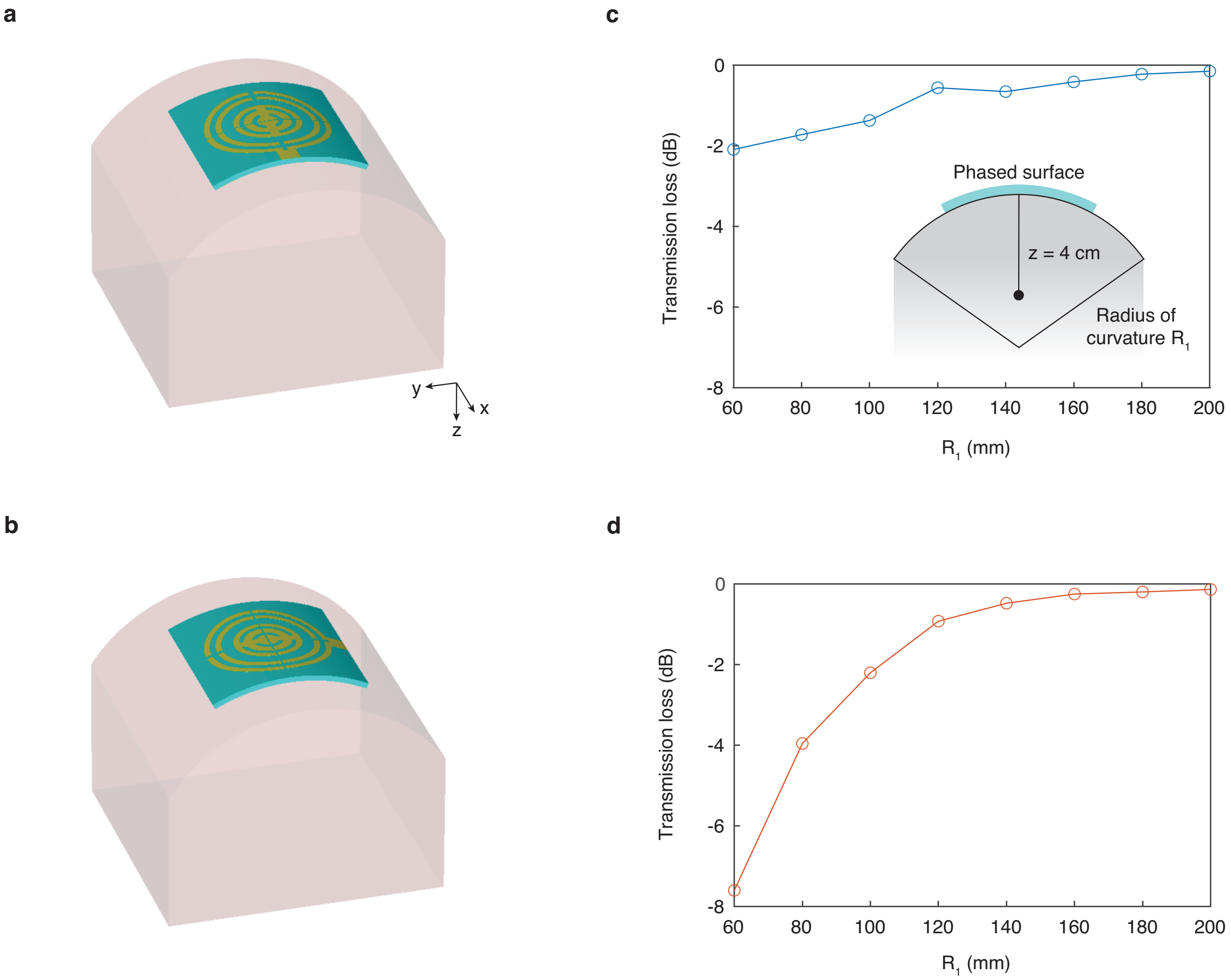

Supplementary Figure 11. Effect of curvature on the transmission efficiency of the side-fed phased surface. **a,** Illustration of the side-fed phased surface on an interface with varying radii-of-curvature R_1. **b,** Illustration of the side-fed phased surface rotated by 90°. **c-d,** Simulated transmission loss of the phased surface caused by bending as a function of R_1 with a dipole antenna placed at $z = 4$ cm.

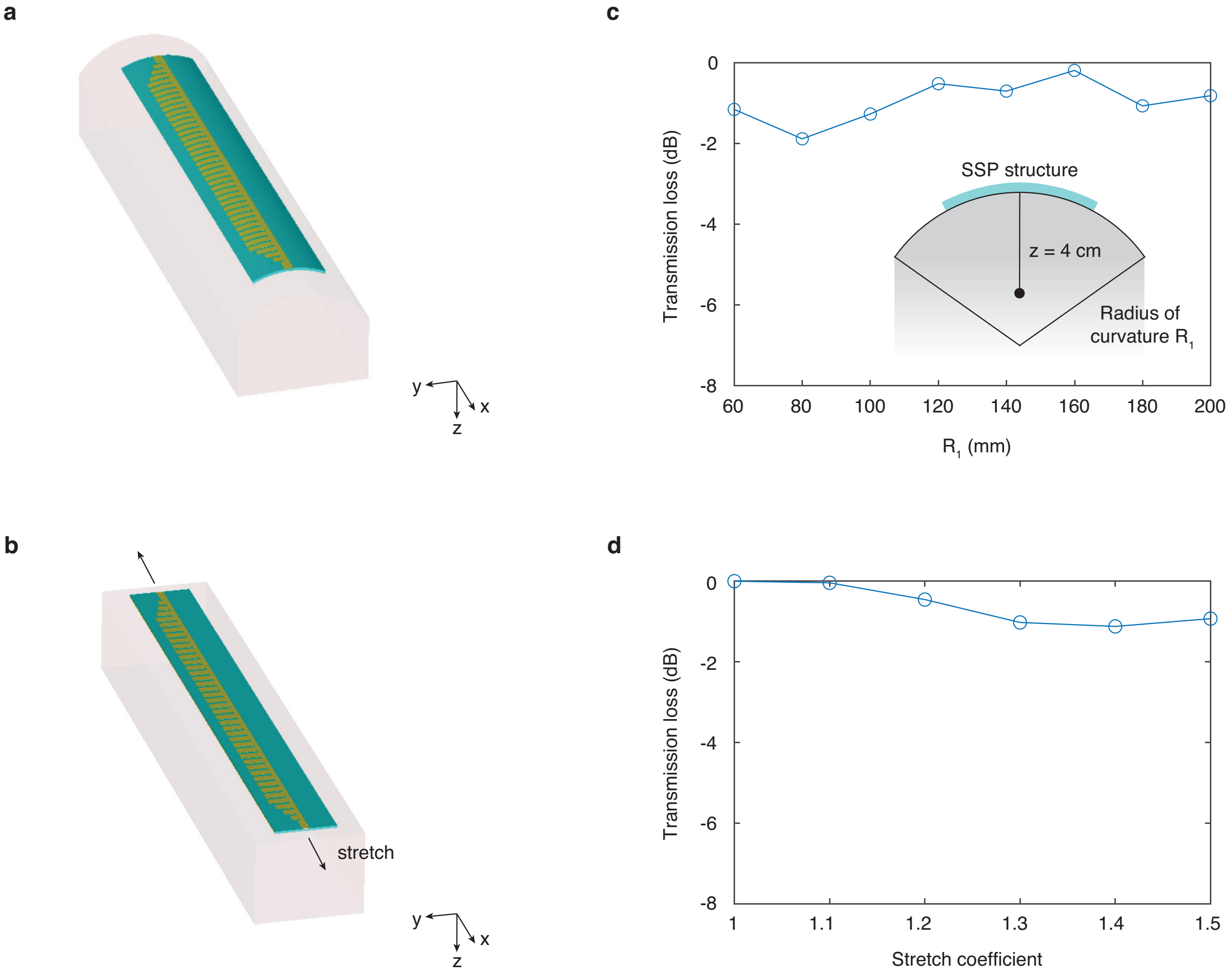

Supplementary Figure 12. Performance of SSP waveguide under different deformations. a-b, Illustration of the SSP waveguide under bending and stretching. **c,** Transmission loss caused by bending as a function of radii-of-curvature R_1. **d,** Transmission loss as a function of the stretch coefficient.

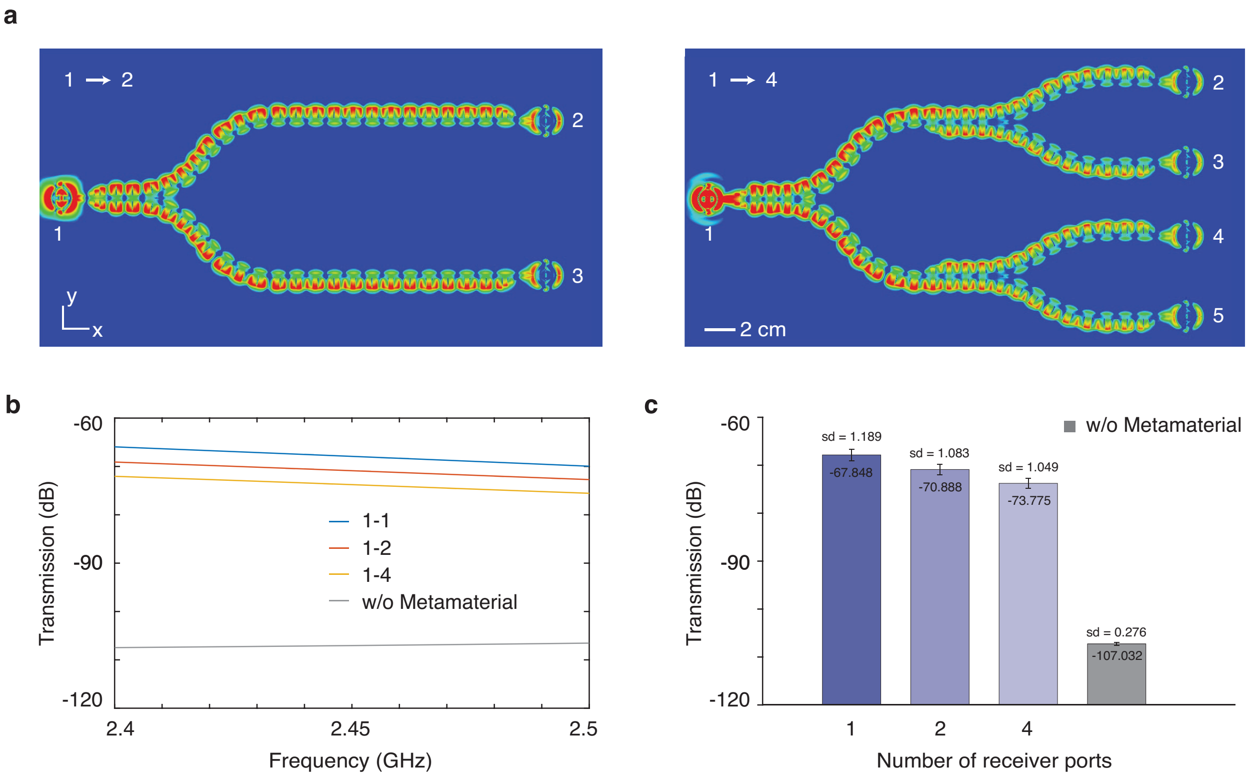

Supplementary Figure 13. Propagation on metamaterial textiles as a power divider. **a,** Top-down projections of simulated magnetic field for the metamaterial textile (2.4 GHz). 2-way and 4-way power dividers were demonstrated. **b,** Transmission coefficient as a function of numbers of receiver ports. **c,** Transmission ($|S_{21}|$) as a function of number of receiver ports. The Grey bar shows transmission measured without the metamaterial textile. Error bars show mean ± s.d. of the transmission spectra in 2.4–2.5 GHz.

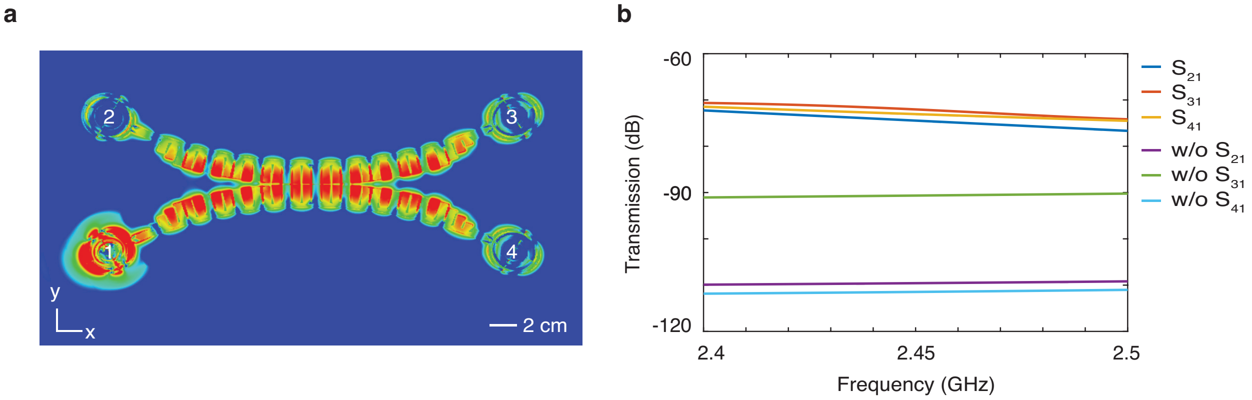

Supplementary Figure 14. Wireless interconnection of four implantable devices with metamaterial textiles. a, Full-wave simulations of the magnetic field (xz plane) at 2.4 GHz for two curved metamaterial textiles. **b,** Transmission efficiency between the transmitter (Implant 1) and receivers (Implant $2-4$) with and without the metamaterial textiles.

a

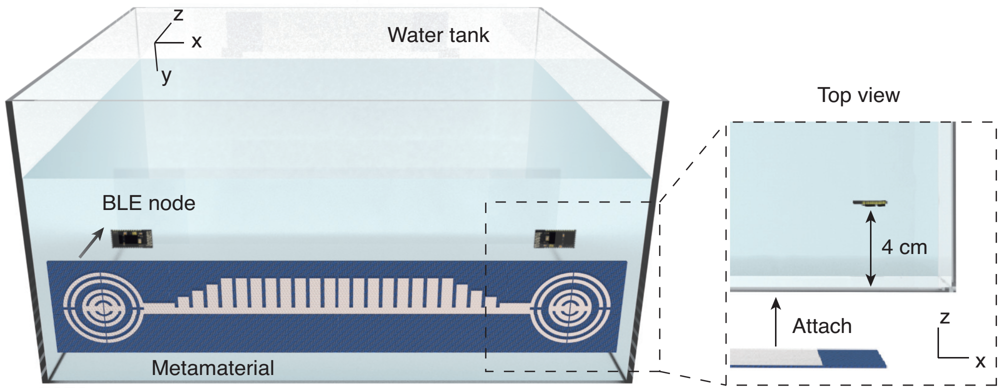

b

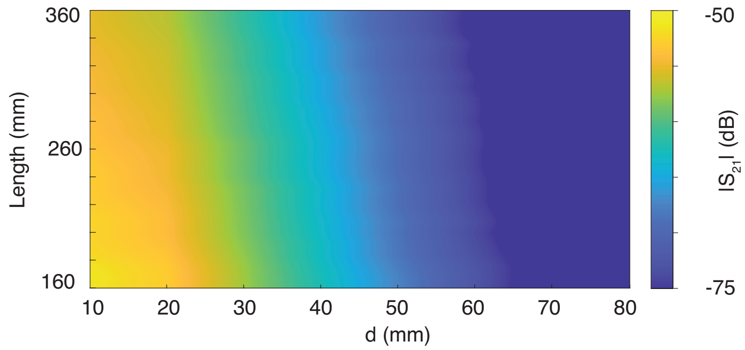

Supplementary Figure 15. Bench-top implant-to-implant wireless communication characterisation. **a,** Illustration of the water container experiment set-up. The metamaterial textile was attached to the wall of the water container. Two implantable BLE modules were fixed at a 4 cm distance. **b,** Simulated transmission coefficient ($|S_{21}|$) as a function of the length of the metamaterial textile and the depth of the implantation.

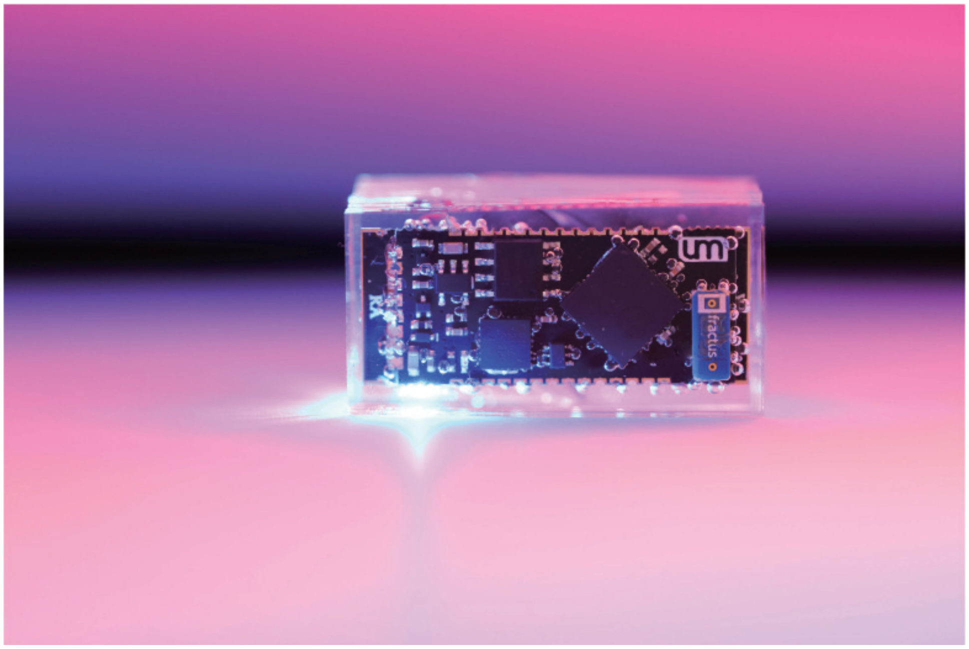

Supplementary Figure 16. Photo of implant node. a, Photograph of an encapsulated implant node (with a LED indicator) used for system characterization.

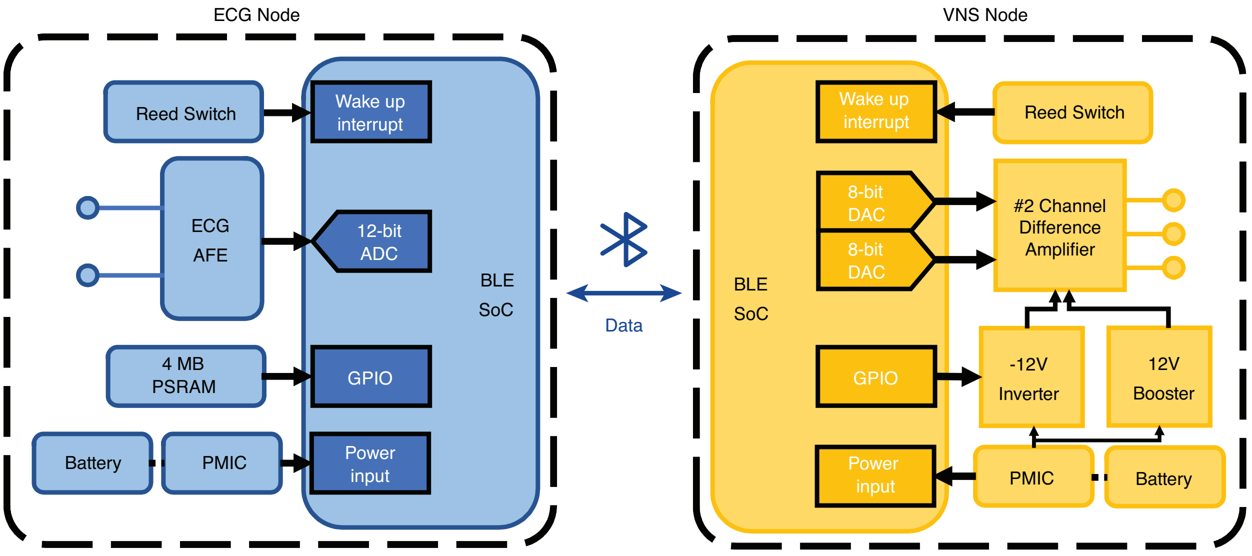

Supplementary Figure 17. Design of implant devices. Block diagrams of system operation of the ECG node (Left) and VNS node (Right) and their wireless communication interface via BLE.

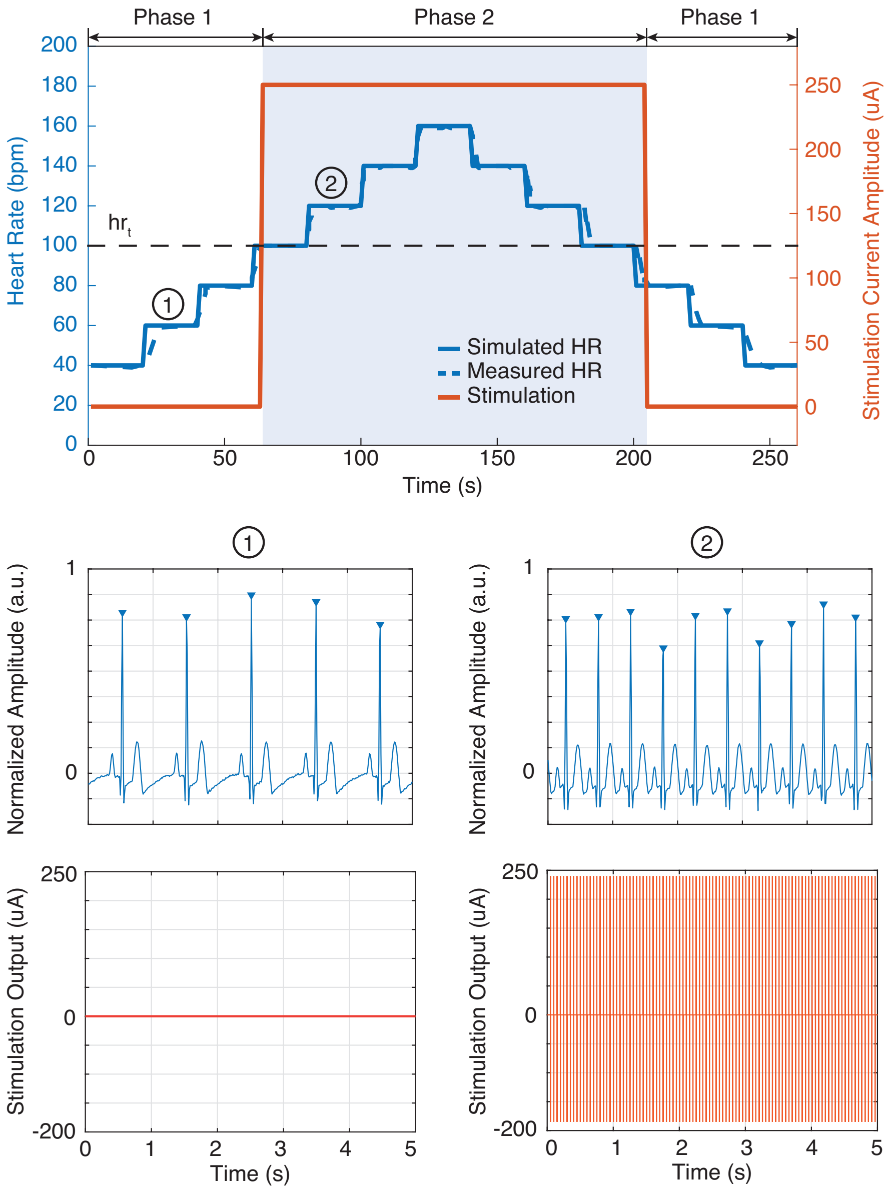

Supplementary Figure 18. Bench-top closed-loop control of implant devices via BLE. Recorded heart rate calculated by the ECG node when connected to an ECG simulator with increased output heart rate. At phase 2, the VNS node was triggered as the heart rate was higher than the preset threshold.

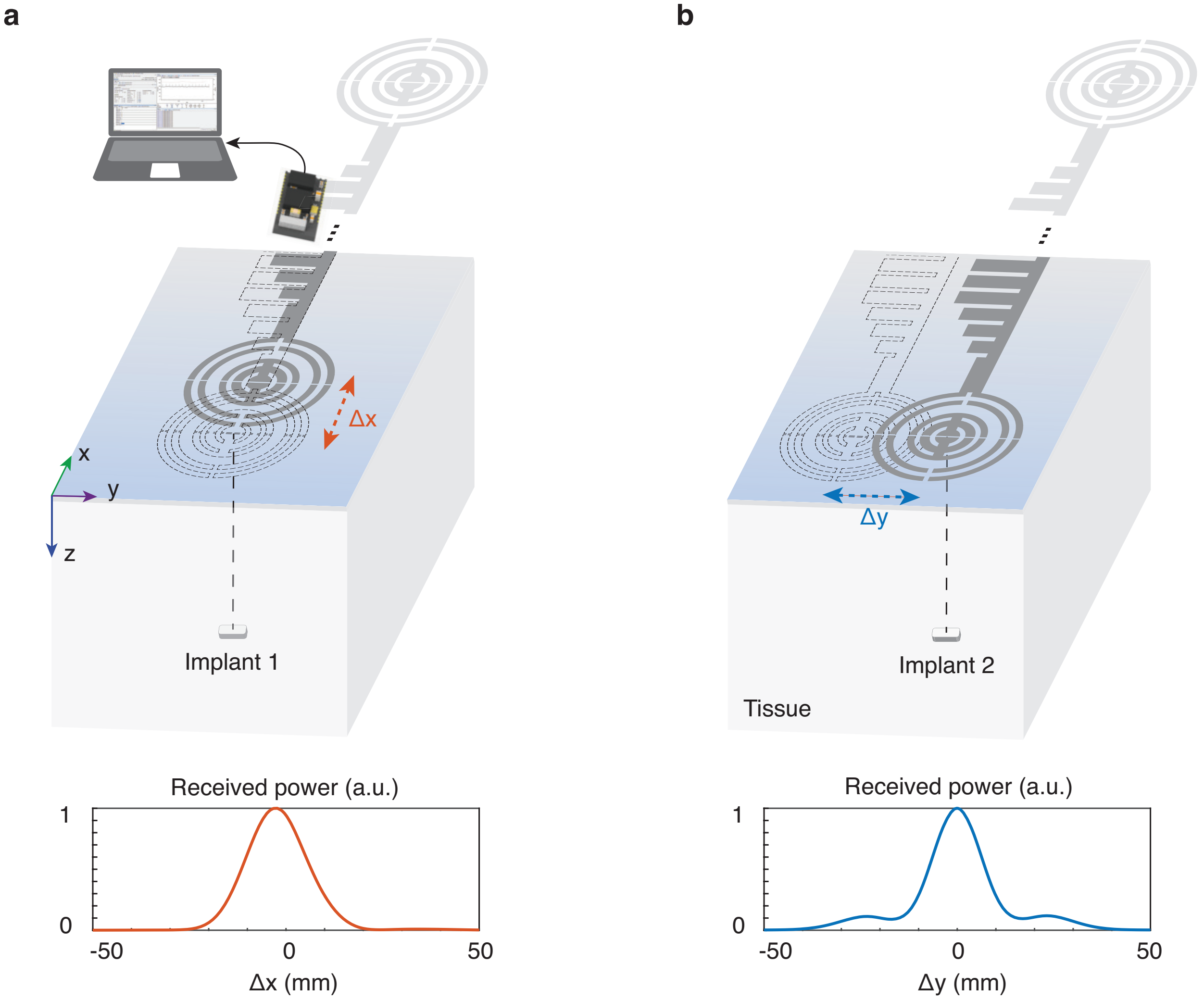

Supplementary Figure 19. Illustration of metamaterial textile placement and alignment. Implantable devices are placed under thick porcine tissue (∼ 3 cm). The metamaterial textile is aligned with the implanted devices in both x (**a**) and y (**b**) directions based on RSSI measurement of an external BLE module.

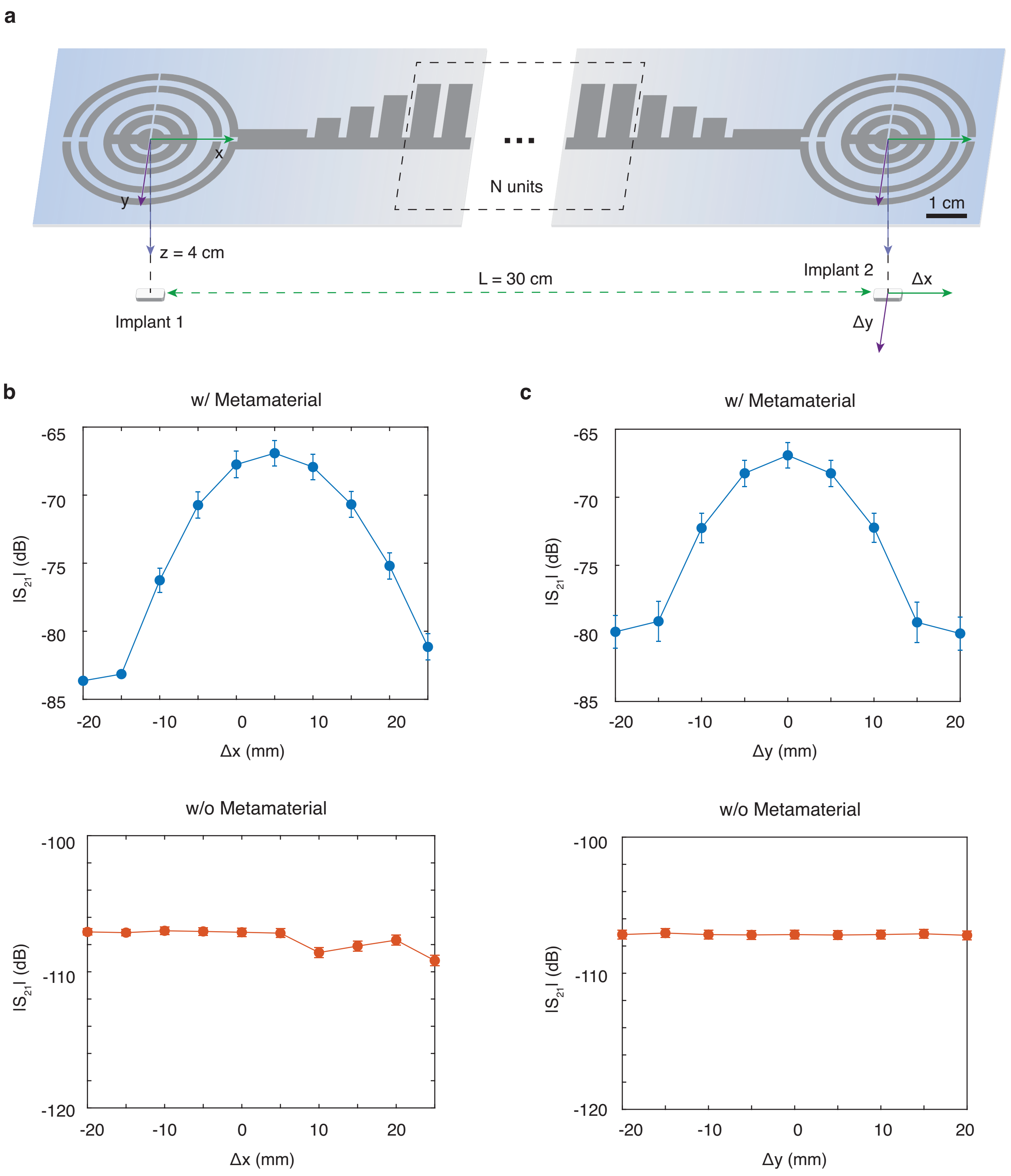

Supplementary Figure 20. Dependence of communication efficiency on displacement of the receiver antenna. a, Schematic of the dipole receiver displacements along x-axis and y-axis. **b-c,** Transmission efficiency ($|S_{21}|$) change as a function of displacement in Δx and Δy. Error bars show mean ± s.d. of the transmission spectra in 2.4–2.5 GHz.

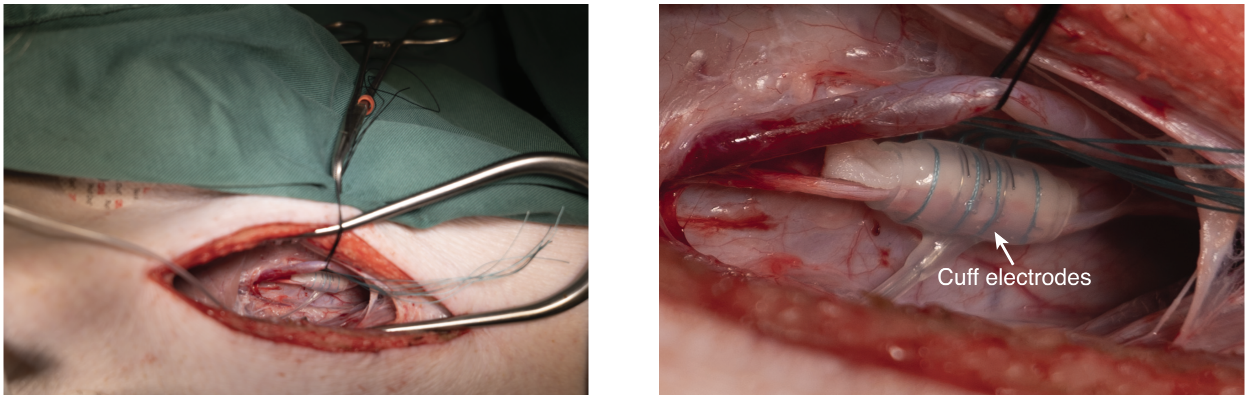

Supplementary Figure 21. In vivo operation of wireless implant devices. Images of surgical procedures for implantation of VNS node in the right side neck area of a pig model. The cuff electrodes were attached to the right cervical vagus nerve.

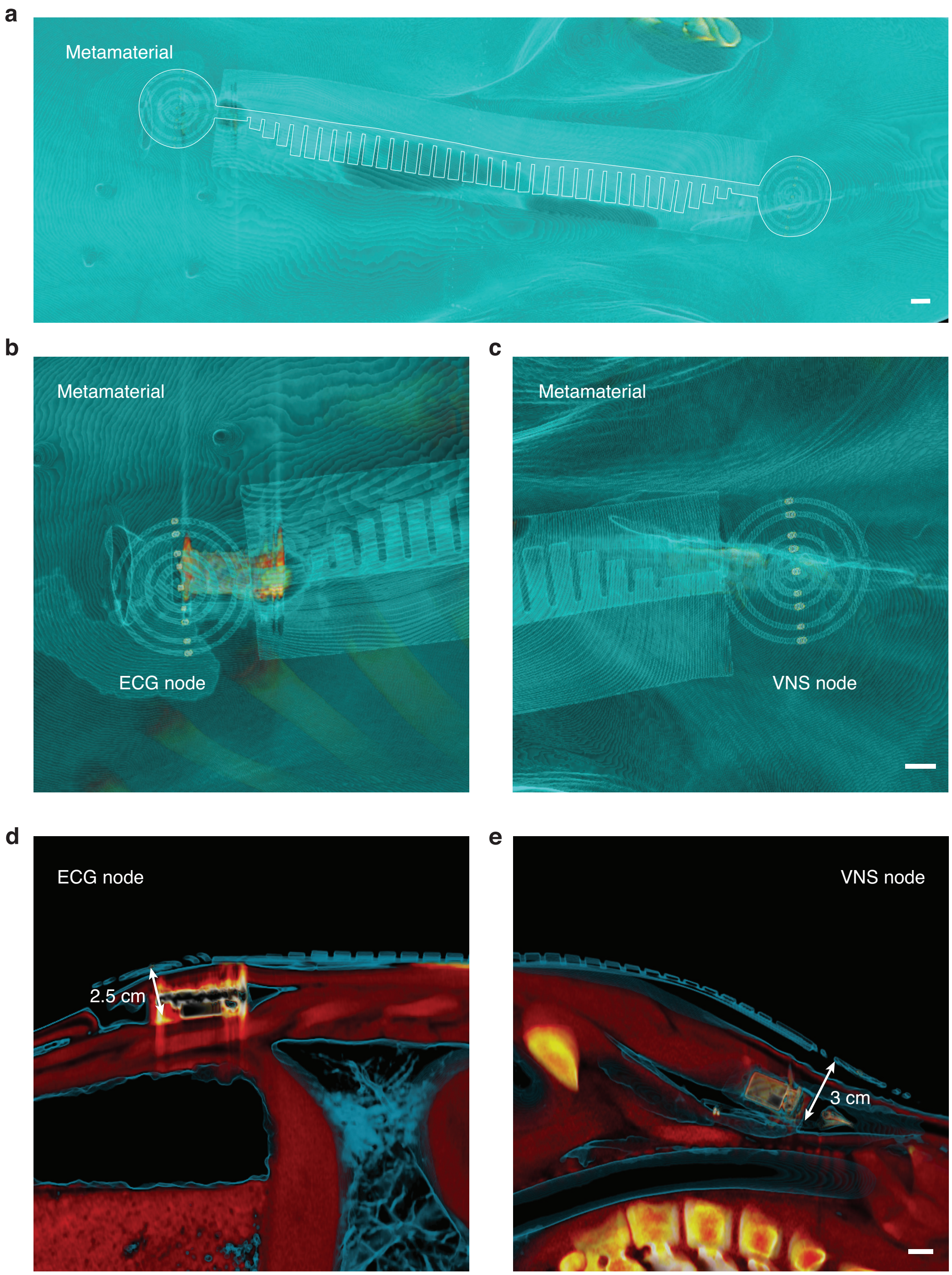

Supplementary Figure 22. In vivo closed-loop control of implantable devices. a-c, Top view of the metamaterial textile placed over the skin of the pig model. **d-e,** Three-dimensional computed tomography images showing the relative position of the metamaterial textile and the implanted ECG and VNS nodes. Scale bar, 1 cm.

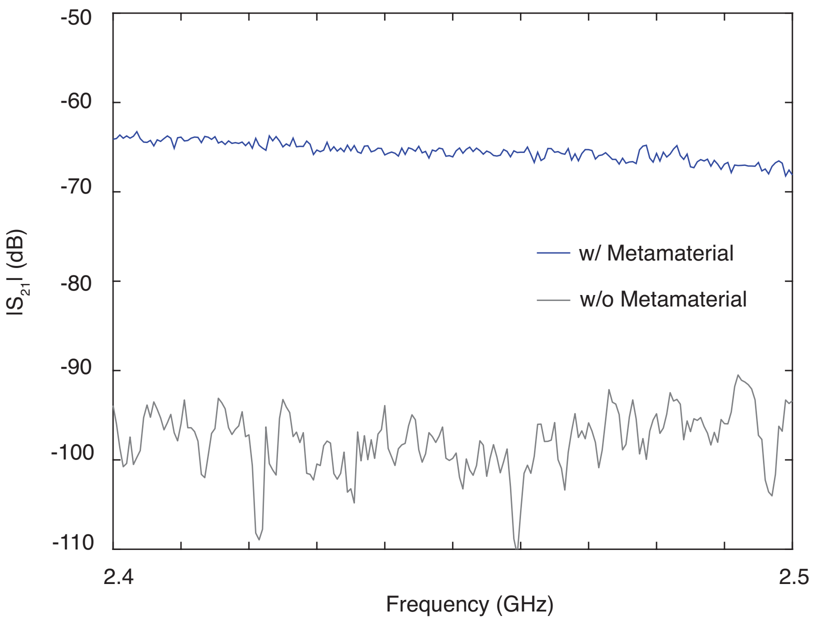

Supplementary Figure 23. Transmission spectrum of implantable devices. Transmission spectrum $|S_{21}|$ between the implant antennas with and without the metamaterial textiles.

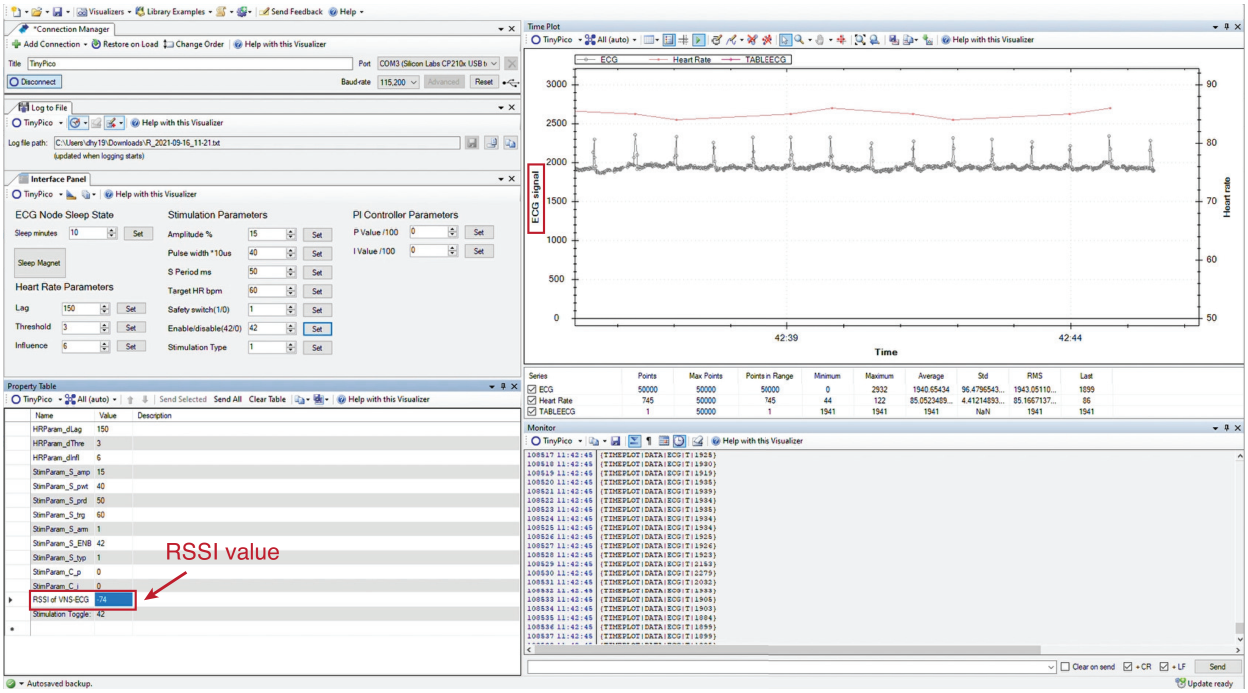

Supplementary Figure 24. System operation. Wireless user interface monitors the implant-to-implant communication using an outside Bluetooth module connected to a computer. To record parameters communicating between the two implanted nodes together with the ECG signal, it first connects to the ECG node (master device) in slave mode. During the monitoring and recording session, parameters used for HR calculation and stimulation control can be reassigned to the ECG node using the interface via BLE.

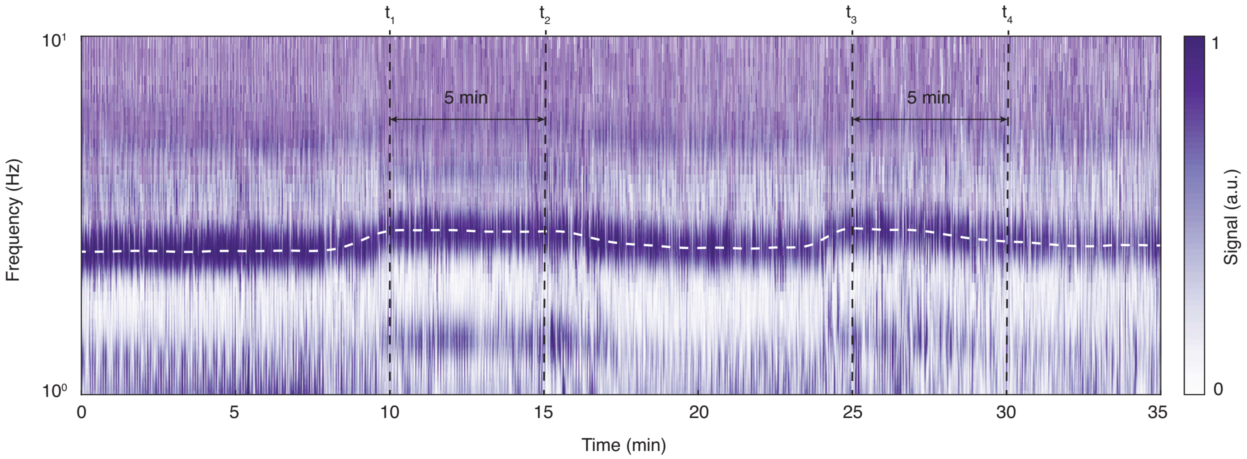

Supplementary Figure 25. In vivo ECG signal monitoring. Spectrogram of recorded ECG signals during two phenylephrine injection cycles.

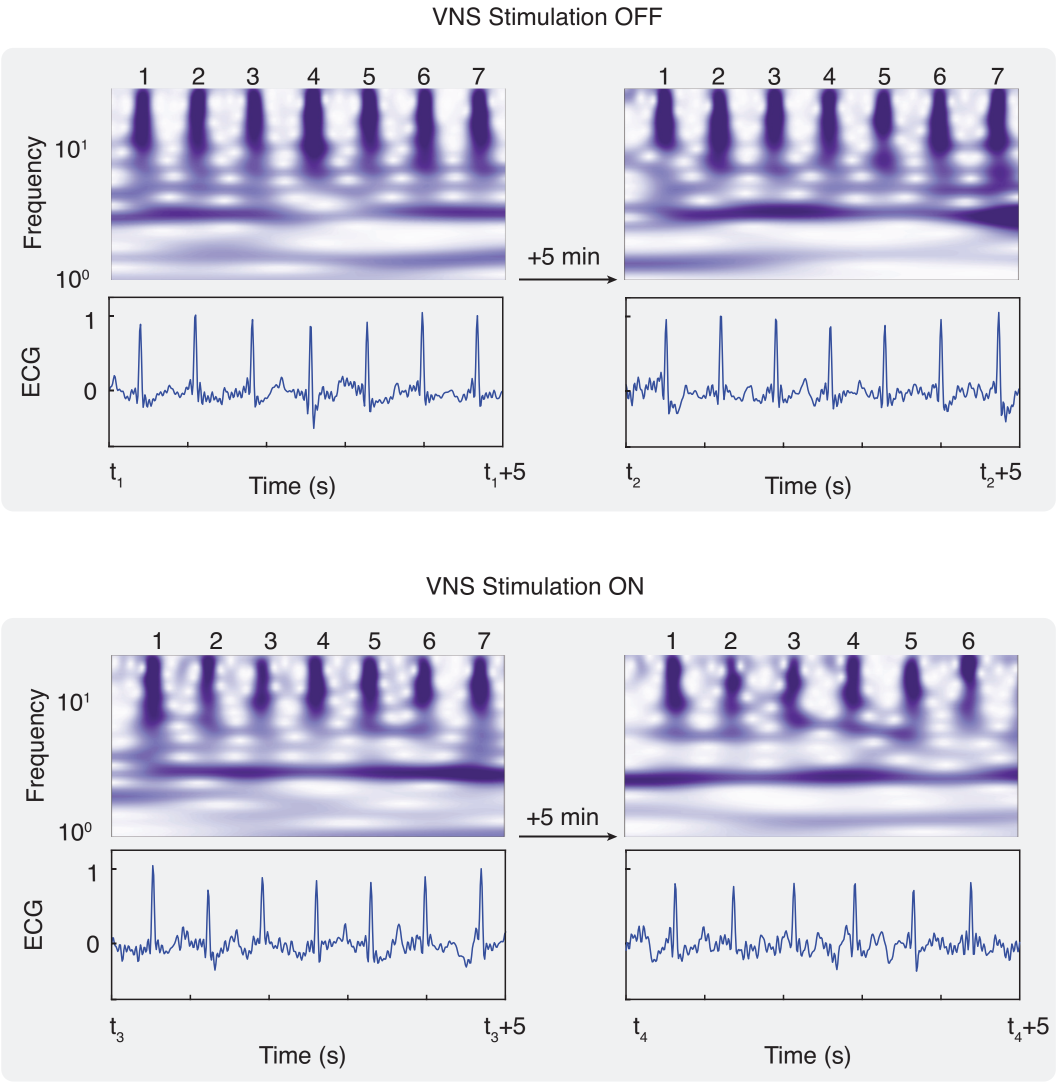

Supplementary Figure 26. Comparison of 5-s spectrogram. Comparison of the spectrogram of ECG signal segment 5 min after the heart rate is plateaued. Fewer heartbeats detected in 5 s indicate lower heart rate.

Supplementary Table 1. Components of the textile-integrated phased surface.

Component	Discripthion	Company	Parrt No
C1	Capacitor (0.8 pF)	Murata Electronics	GJM1555C1HR80BB01D
C2	Capacitor (0.4 pF)	Murata Electronics	GJM1555C1HR40BB01D
C3	Capacitor (0.3 pF)	Murata Electronics	GJM1555C1HR30BB01D
C4	Capacitor (0.7 pF)	Murata Electronics	GJM1555C1HR70BB01D
C5	Capacitor (4.0 pF)	Murata Electronics	GJM1555C1H4R0BB01D
