# Supplementary material for: Implant-to-implant wireless networking with metamaterial textiles
Source: Nat Commun. 2023 Jul 19;14:4335. doi: 10.1038/s41467-023-39850-2 (PMC10356940; doi:10.1038/s41467-023-39850-2)
Supplement: Supplementary file 3 — Description of Additional Supplementary Files [file 41467_2023_39850_MOESM3_ESM.pdf]

<u>Description of Additional Supplementary Files</u>

File Name: Supplementary Movie 1

Description: **Benchtop demonstration of wireless implant communications with metamaterial textiles.** Two Bluetooth modules are placed inside an acrylic container while the relative signal strength indicator (RSSI) between the two modules is recorded in real-time. Initially, the two modules are connected as the container is empty. After pouring water into the container and the two modules are fully submerged, the RSSI is dropped to -100 dB, denoting a disconnection. Placing the metamaterial textile on the wall of the container re-establish the wireless connection between the two modules. Repeatedly placing and removing the metamaterial textile shows the capability of our metamaterial textiles to enable wireless implant communications.
